# Comparison of stress distribution around splinted and nonsplinted implants with different crown height space in posterior mandible: A finite element analysis study

**DOI:** 10.1111/jopr.13935

**Published:** 2024-09-03

**Authors:** Hamiyet Güngör Erdoğan, Mert Keleş, Burak Yılmaz

**Affiliations:** ^1^ Department of Prosthetic Dentistry Lokman Hekim University Ankara Turkey; ^2^ Department of Periodontology Lokman Hekim University Ankara Turkey; ^3^ Department of Reconstructive Dentistry and Gerodontology School of Dental Medicine, University of Bern Bern Switzerland; ^4^ Department of Restorative, Preventive and Pediatric Dentistry School of Dental Medicine, University of Bern Bern Switzerland; ^5^ Division of Restorative and Prosthetic Dentistry The Ohio State University Columbus Ohio USA

**Keywords:** crown height space, dental implants, finite element analysis, nonsplinted, splinted

## Abstract

**Purpose:**

The purpose of this finite element analysis (FEA) study was to analyze the stress distribution on prosthetic components of splinted and nonsplinted prostheses, bone, and implants with different crown height space (CHS).

**Materials and Methods:**

Mandibular posterior segment was modeled with no resorption at the second premolar site and various amounts of resorption (0, 3, 6, and 9 mm) at the first molar site. Two adjacent implants (Straumann bone level implants, 4.1 mm×8 mm) were placed; at the second premolar site, the crown height was 8 mm and at the first molar site, the crown height varied (8, 11, 14, and 17 mm), depending on the amount of resorption. Both splinted and nonsplinted crowns were designed. Vertical and oblique loads of 400 N were applied to the crowns. von Mises stress was used to evaluate the stress distribution in the implant complex and maximum principal stress was used to evaluate the stress in the bone.

**Results:**

When oblique forces were applied, the highest von Mises stresses were observed for nonsplinted crowns in the 17 mm CHS group. The maximum principal and minimum principal stresses observed in bone under oblique loading increased with increased CHS for nonsplinted restorations.

**Conclusions:**

Crown height affected the amount of stress in bone and implant components. When the crown height difference between two adjacent implants increases, splinting may be crucial.

Dental implants have high long‐term success rates in the treatment of complete and partial edentulism.^1^, ^2^ However, the treatment of the posterior partially edentulous mandible with dental implants may present several challenges, particularly when there is severe bone resorption due to early and traumatic tooth extractions and hard tissue augmentation procedures may be required.^3^ However, augmentation procedures may require long healing times and a secondary surgical intervention at another region can be costly and also unpredictable as they may result in infection and lead to the loss of graft material. Moreover, systemic disease, age, and smoking habits are some of the surgical limitations.^4^ With the presence of severe bone loss and when augmentation cannot be performed, short implants and increased crown height are inevitable.^5^ The results of in vitro studies pointed out that care is needed in the presence of increased crown height, as it is one of the major reasons for excessive occlusal loads with an increased moment arm.^6^ Particularly under oblique forces, a high crown‐to‐implant ratio (CIR) can negatively affect dental implants and bone by contributing to stress accumulation.^5^, ^7^ The other concept named crown height space (CHS) is just as important as CIR. CHS is defined as the distance between the crest of the alveolar bone and the occlusal table.^8^ With increased CHS, oblique loads can result in excessive lateral movement leading to stress concentration at the implant platform.^9^

The resulting stresses can shorten the lifespan of dental implants and increase the risk of prosthetic complications such as recurrent abutment screw loosening and porcelain fracture.^10^, ^11^ Splinting of crowns on adjacent implants is thought to help evenly distribute the occlusal forces on implants. Thus, potential overloading of the crestal bone may be minimized, which can decrease the incidence of complications such as prosthetic failure, implant fracture, and implant failure.^12^, ^13^ However, splinted crowns can interfere with proper oral hygiene care and complicate clinical and laboratory procedures.^14^ There are studies in the literature comparing the load distribution of splinted and nonsplinted implants.^15^, ^16^, ^17^ Some studies have shown that splinting of implants decreased the stress on the mandible and changed the stresses on the restoration.^18^, ^19^ However, there are also other studies reporting that splinting increased the maximum stresses on the mandible.^16^

Strain gauges, photoelasticity, three‐dimensional (3D) digital image correlation, and finite element analyses (FEA) are the methods that have been used to evaluate the stress and strain distribution of the implant complex.^20^, ^21^, ^22^ While FEA creates solid and complex objects with precision, it is an engineering method that calculates stresses and strains inside an object. The method allows the application of different occlusal forces, and resulting strains and stresses can be exported as images and as quantitative data.^23^, ^24^

Studies reported conflicting findings for the effect of splinting on stress distribution for crowns, and a consensus on this matter has not been reached yet.^12^, ^16^, ^25^ A review of the literature revealed that when evaluating the stresses occurring in two splinted or nonsplinted implants, the CHS for two adjacent implants was assumed to be the same. However, two adjacent implants may have different CHS due to potential differences in crestal bone levels depending on extractions that were performed at different times. In addition, in a previous study, it was reported that prosthetic complications increased when the CHS of splinted implant crowns was more than 15 mm.^10^ However, how the strains develop when splinted or nonsplinted adjacent implant crowns have a CHS of more than 15 mm has not been reported in the literature. The aim of this study was to model two adjacent implants in a partially edentulous posterior mandible and splinted and nonsplinted restorations with different CHS, and to evaluate the effect of splinting and CHS on the stresses at bone, implant, and abutment screw with 3D FEA. The null hypotheses of this study were that the CHS and splinting would not affect the stresses measured at bone, implant, and abutment screw.

## MATERIALS AND METHODS

**FIGURE 1:**
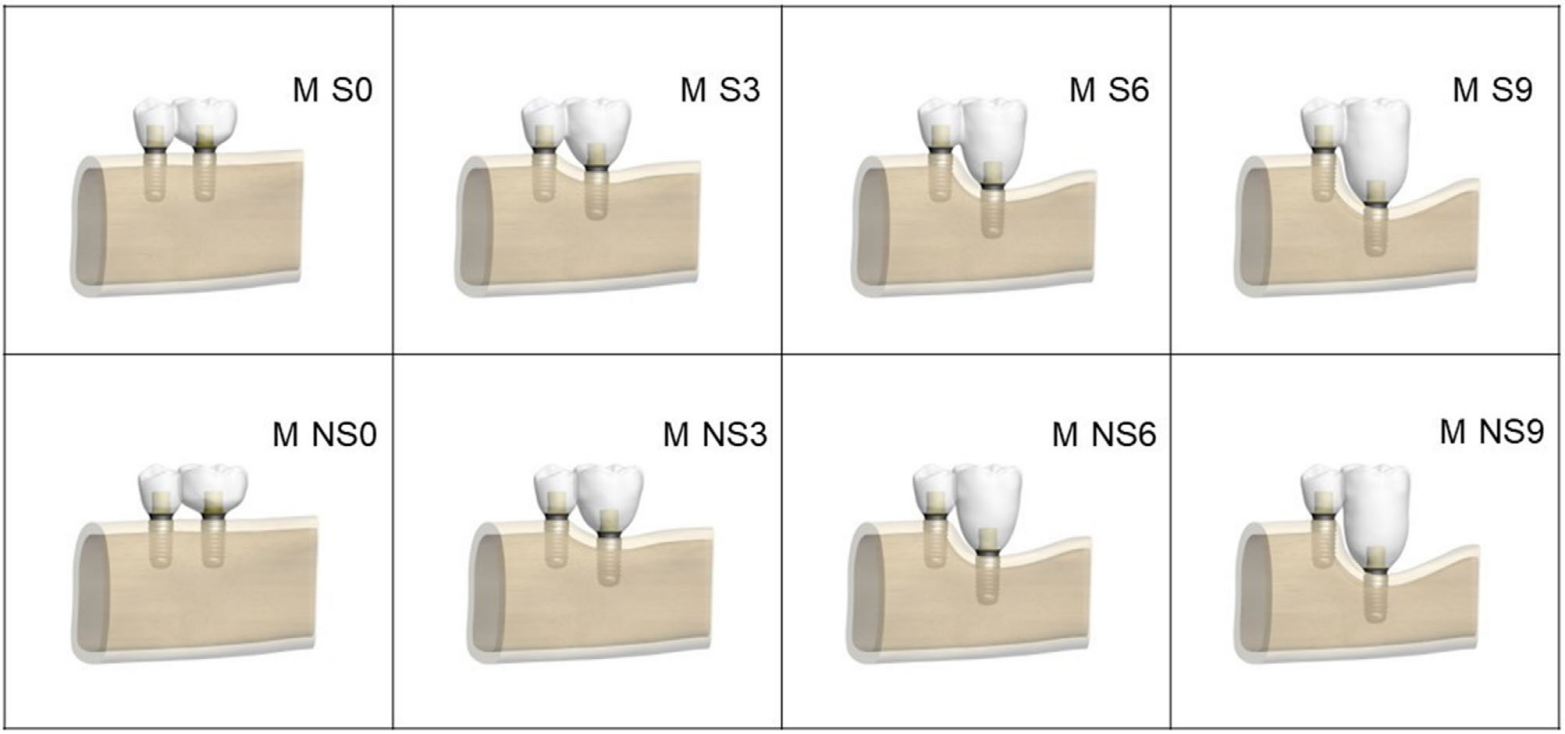
Reference models of splinted and nonsplinted implants with different crown height space at first molar site.

In the present study, eight 3D finite element models were constructed (Figure 1). Models were designed based on four different bone resorption levels (0, 3, 6, and 9 mm) and two different prosthetic designs (splinted and nonsplinted) (Table 1). Two identical (8 mm in height and 4.1 mm in diameter) implants (Straumann bone level implants; Institut Straumann AG, Basel, Switzerland) were placed on the second premolar site and the first molar site in the posterior mandible (Figure 1). The acquisition of bone data from tomography data was performed with 3D Slicer software program (http://www.slicer.org), while the solid models were made suitable for the analysis environment and the optimized mesh was created with a finite element software program (ANSYS Workbench 18.2.2; ANSYS Inc, Pennsylvania, USA). LS‐DYNA solver was used to solve the FEA models. Each model consisted of the implant complex and the bone structure of the mandibular molar region. In the modeled bone structure, a 2 mm layer of cortical bone was designed around the cancellous bone.^26^ The bone level was constant at the second premolar site, while in the first molar site, 0, 3, 6, and 9 mm resorption situations were simulated. While the crown height was 8 mm for each model at the second premolar site, it was 8, 11, 14, and 17 mm at the first molar site in accordance with the amount of resorption. To evaluate the stress on the bone and prosthetic components caused by two adjacent implants with different bone levels, splinted and nonsplinted superstructures were designed from monolithic zirconia material based on Wheeler atlas data. A type of titanium base abutment (Regular CrossFit Variobase for Crown; Institut Straumann AG, Basel, Switzerland) of 4.5 mm diameter, 1 mm gingival height, 3.5 mm height, and abutment screw (Regular CrossFit Basal Screw for crown; Institut Straumann AG, Basel, Switzerland) were designed for nonsplinted crowns whereas another type of titanium base abutment (Regular CrossFit Variobase for bridge/bar; Institut Straumann AG, Basel, Switzerland) of 4.5 mm diameter, 1 mm gingival height, 3.5 mm height and abutment screw (Regular CrossFit Screw‐Retained Bars and Bridges Bone Level Screw for splinted crown; Institut Straumann AG, Basel, Switzerland) were designed for splinted crowns. The connector area modeled in the splinted design 4 × 2.5 mm^2^, increased by 1 mm in each model with increasing CHS.^27^ The implant complex consisted of crown, abutment, abutment screw, and implant. Material properties were determined according to the properties reported in previous studies (Table 2). All materials are assumed to be linearly elastic, homogeneous, and isotropic.

**TABLE 1:** Model descriptions.

Prosthetic design	Bone resorption		Models	Nodes/elements
	FMS	SPS		
Splinted crowns	0 mm	0 mm	Model S0 (M S0)	310630/1231344
	3 mm	0 mm	Model S3 (M S3)	1213333/4933535
	6 mm	0 mm	Model S6 (M S6)	1211737/4923890
	9 mm	0 mm	Model S9 (M S9)	1196450/4853646
Nonsplinted crowns	0 mm	0 mm	Model NS0 (M NS0)	1071894/4324493
	3 mm	0 mm	Model NS3 (M NS3)	1056480/4263992
	6 mm	0 mm	Model NS6 (M NS6)	1066140/4295039
	9 mm	0 mm	Model NS9 (M NS9)	1051560/4221064

Abbreviations: FMS, first molar site; NS, nonsplited; S, splinted; SPS, second premolar site.

**TABLE 2:** Material properties used in finite element models.

Materials	Modulus of elasticity (MPa)	Poisson ratio (v)	References
Cortical bone	13,700	0.3	Bulaqi et al.^23^ Lemos et al.^28^
Trabecular bone	1370	0.3	Bulaqi et al.^23^ Lemos et al.^28^
Titanium	110,000	0.35	Bulaqi et al.^23^ Lemos et al.^28^
Zirconia	210,000	0.3	Franco‐Tabares et al.^29^ Miura et al.^30^

**FIGURE 2:**
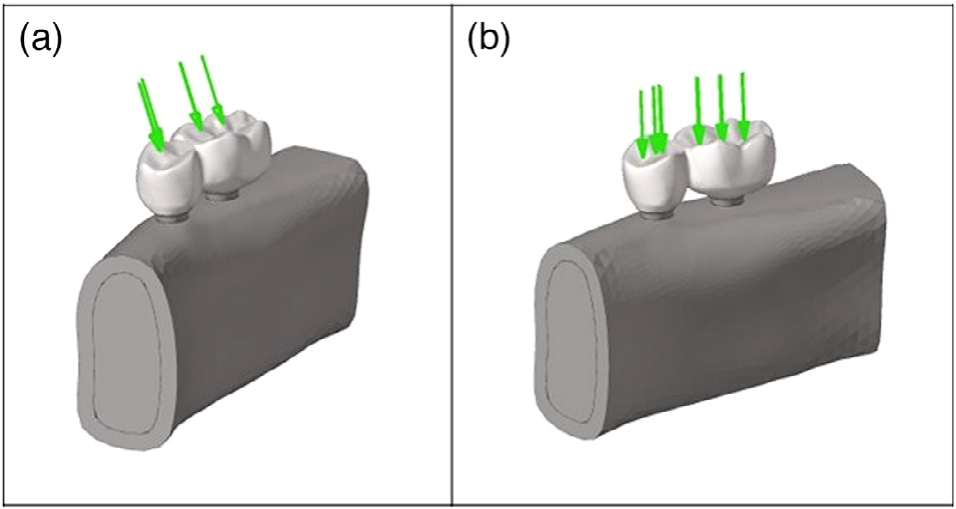
Applied loads.

A total oblique force of 400 N (200N×2) was applied to the lingual slopes of the buccal cusps of the premolar and molar teeth (2 points per tooth) in the buccolingual direction at an angle of 30 degrees (Figure 2a). A total vertical force of 400 N (200N×2) was applied to the fossa of premolar and molar teeth (3 points per tooth) in a perpendicular direction (Figure 2b).

The models were fixed at the nodes located in the anterior, posterior, and inferior regions of the bone by restricting all degrees of freedom to prevent movement in all three axes. A total of 16 linear static analyses were performed for eight analysis models under the specified force and boundary conditions.

To be able to perform the analyses in the mathematical models created and to obtain accurate results, the surface relations of the parts forming the model with each other must be defined in the analysis program. In all analyses, a bonded type of contact definition was made between the contacting components. This approach assumes that the parts move with full correlation during movement. All FEA were performed using FEA software (ABAQUS 6.14; Dassault Systèmes SIMULIA Corp). Von Mises stress values were used to evaluate the stress distribution in the implant and abutment. The maximum and minimum principal stress was evaluated for the bone tissue.

## RESULTS

For all components, von Mises stress values and maximum and minimum principal stress values under applied vertical and oblique forces are given in Tables 3 and 4, respectively. Except for the stresses occurring in the abutment screw of the implant placed at the first molar site under the applied vertical force, the forces decreased slightly as the amount of resorption increased in all components. Whether the models were splinted or nonsplinted had no significant effect on stress distribution.

**TABLE 3:** von Mises Stress (MPa) values of the implant complex under loading conditions.

	Vertical loading						Oblique loading					
	Implant		Abutment		Abutment screw		Implant		Abutment		Abutment screw	
	FMS	SPS	FMS	SPS	FMS	SPS	FMS	SPS	FMS	SPS	FMS	SPS
M S0	75.6	78.9	99.8	105.8	33.4	34.4	246.1	270.9	307.3	352.6	101.8	107.9
M S3	73.6	75	96.3	100	34.5	30.7	251.9	269.7	330.8	347.5	108.1	98.3
M S6	73.3	67.1	95	89.5	34.7	26	253.8	260.1	336.2	345.2	109	79.3
M S9	72.1	60.4	94.4	82.1	34.8	23.6	257.3	238.4	341.9	320.6	110.5	58.1
M NS0	85.7	61.7	124.4	96.2	31.6	21.9	253.1	230.9	354.6	364	85.4	70.5
M NS3	77.1	61.2	121.9	95.8	32.9	21	310.3	230.4	415.4	363.3	120.6	69.2
M NS6	77.1	60.4	119.9	94.5	33.3	20.5	389.3	229.3	499.1	361.8	148	68.7
M NS9	76.3	57.8	114.7	92.3	33.9	20.5	426.7	228	614	360.3	177.3	68.4

Abbreviations: FMS, first molar site; NS, nonsplinted; S, splinted; SPS, second premolar site.

**TABLE 4:** Maximum and minimum principal stress (MPa) in bone under loading conditions.

	Vertical Loading								Oblique Loading							
	Max principal				Min principal				Max principal				Min principal			
	Cortical bone		Cancellous bone		Cortical bone		Cancellous bone		Cortical bone		Cancellous bone		Cortical bone		Cancellous bone	
	FMS	SPS	FMS	SPS	FMS	SPS	FMS	SPS	FMS	SPS	FMS	SPS	FMS	SPS	FMS	SPS
M S0	11.40	9	2.852	2.847	18.5	17.8	−1.180	−1.109	32.7	25	4.061	4.122	42.3	45.7	−1.822	−2.183
M S3	9.8	8.6	1.837	2.710	18.5	17.6	−1.319	−1.056	32.1	27.7	2.287	4.288	41.2	51.6	−2.380	−2.284
M S6	9.6	8.5	1.725	2.479	18.2	17	−1.341	−0.998	31.5	28.1	2.090	4.564	40.5	53.1	−2.417	−2.293
M S9	9.3	8.4	1.718	2.054	17.7	15.7	−1.379	−0.880	30.6	28.2	2.058	4.801	39.7	57.7	−2.552	−2.303
M NS0	11.9	14.6	3.757	3.009	18.7	15.2	−0.942	−0.937	32.7	24.7	4.484	4.084	45.1	45.2	−1.984	−2.124
M NS3	10.7	13.1	2.273	2.847	18.6	14.3	−1.237	−0.905	48.7	24.6	4.495	3.887	67.6	44.7	−3.144	−2.025
M NS6	10.6	12.6	2.098	2.663	18.2	13.9	−1.289	−0.867	57	23.8	4.594	3.428	80.5	44.1	−3.997	−2.004
M NS9	9.9	11.5	2.072	2.289	17.4	12.9	−1.306	−0.748	76.7	19.9	4.987	3.160	93.6	43.1	−4.725	−1.979

Abbreviations: FMS, first molar site; NS, nonsplinted; s, splinted; SPS, second premolar site.

As resorption increased in splinted models under the applied oblique force, von Mises stresses increased in structures such as implant, abutment, and abutment screw in the first molar site, while it remained similar or decreased slightly in these components in the second premolar site (Table 3). The maximum and minimum principal stresses occurring in the bone tissue were not significantly affected by the amount of resorption (Table 4). For the nonsplinted models, the von Mises stresses in the implant, abutment, and abutment screw and the maximum and minimum principal stresses in the bone increased significantly with increased resorption at the first molar site. The increase in resorption at the first molar site did not cause any change in the components at the second premolar site. The splinting substantially minimized the stress, especially in the resorption zone.

**FIGURE 3:**
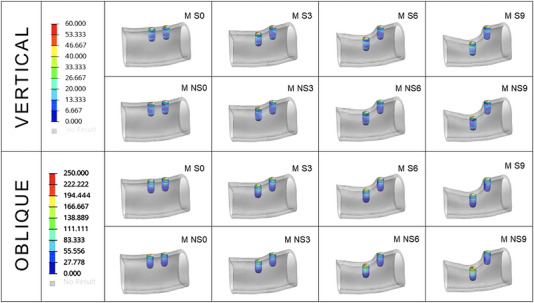
The von Mises stress distribution on implants under vertical and oblique loading. (M S0) Splinted and 0 mm bone resorption, (M S3) splinted and 3 mm bone resorption, (M S6) splinted and 6 mm bone resorption, (M S9) splinted and 9 mm bone resorption. (M NS0) Nonsplinted and 0 mm bone resorption, (M NS3) nonsplinted and 3 mm bone resorption, (M NS6) nonsplinted and 6 mm bone resorption, (M NS9) nonsplinted and 9 mm bone resorption.

**FIGURE 4:**
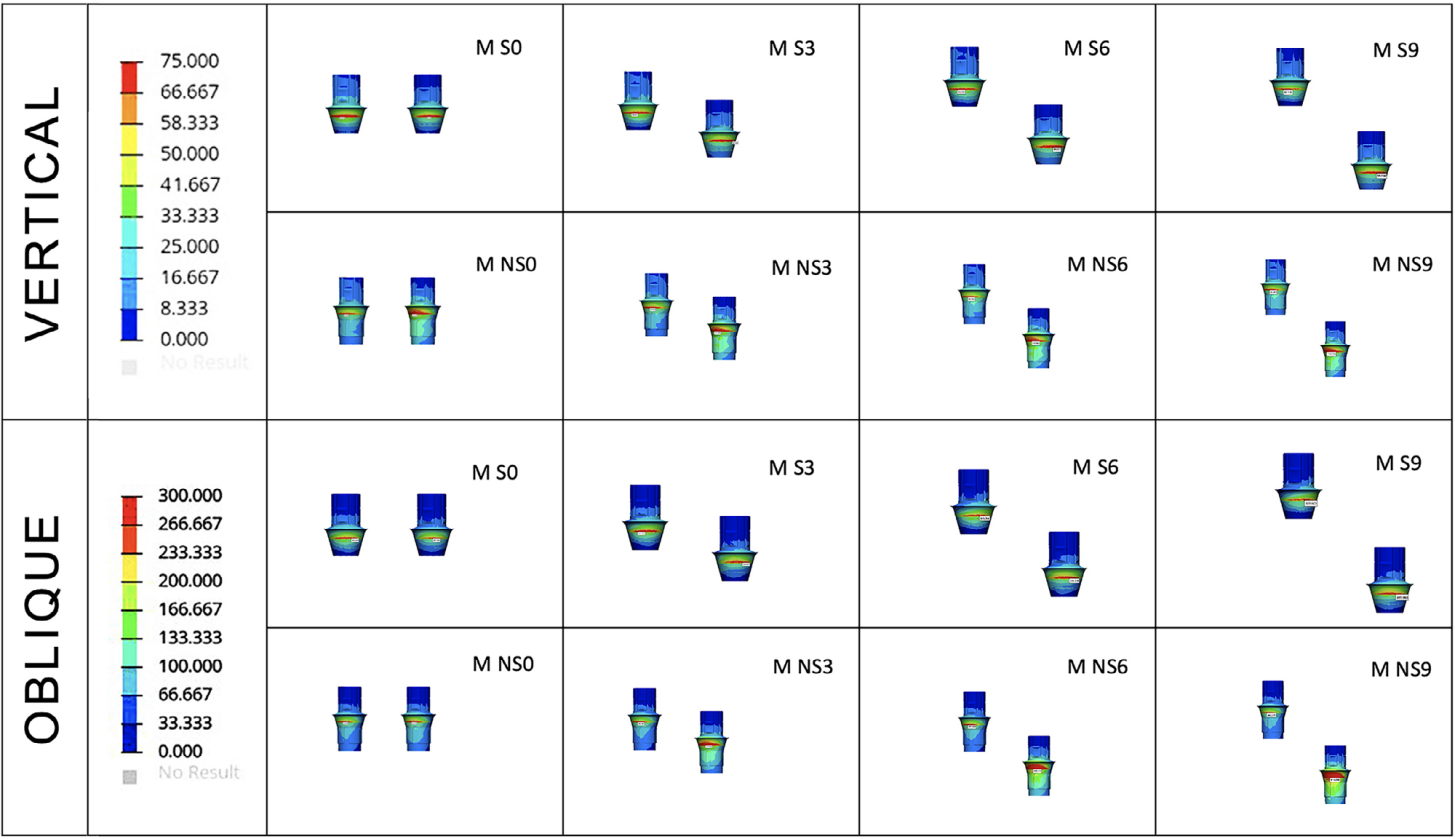
The von Mises stress distribution on titanium base abutment under vertical and oblique loading. (M S0) Splinted and 0 mm bone resorption, (M S3) splinted and 3 mm bone resorption, (M S6) splinted and 6 mm bone resorption, (M S9) splinted and 9 mm bone resorption. (M NS0) Nonsplinted and 0 mm bone resorption, (M NS3) nonsplinted and 3 mm bone resorption, (M NS6) nonsplinted and 6 mm bone resorption, (M NS9) nonsplinted and 9 mm bone resorption.

**FIGURE 5:**
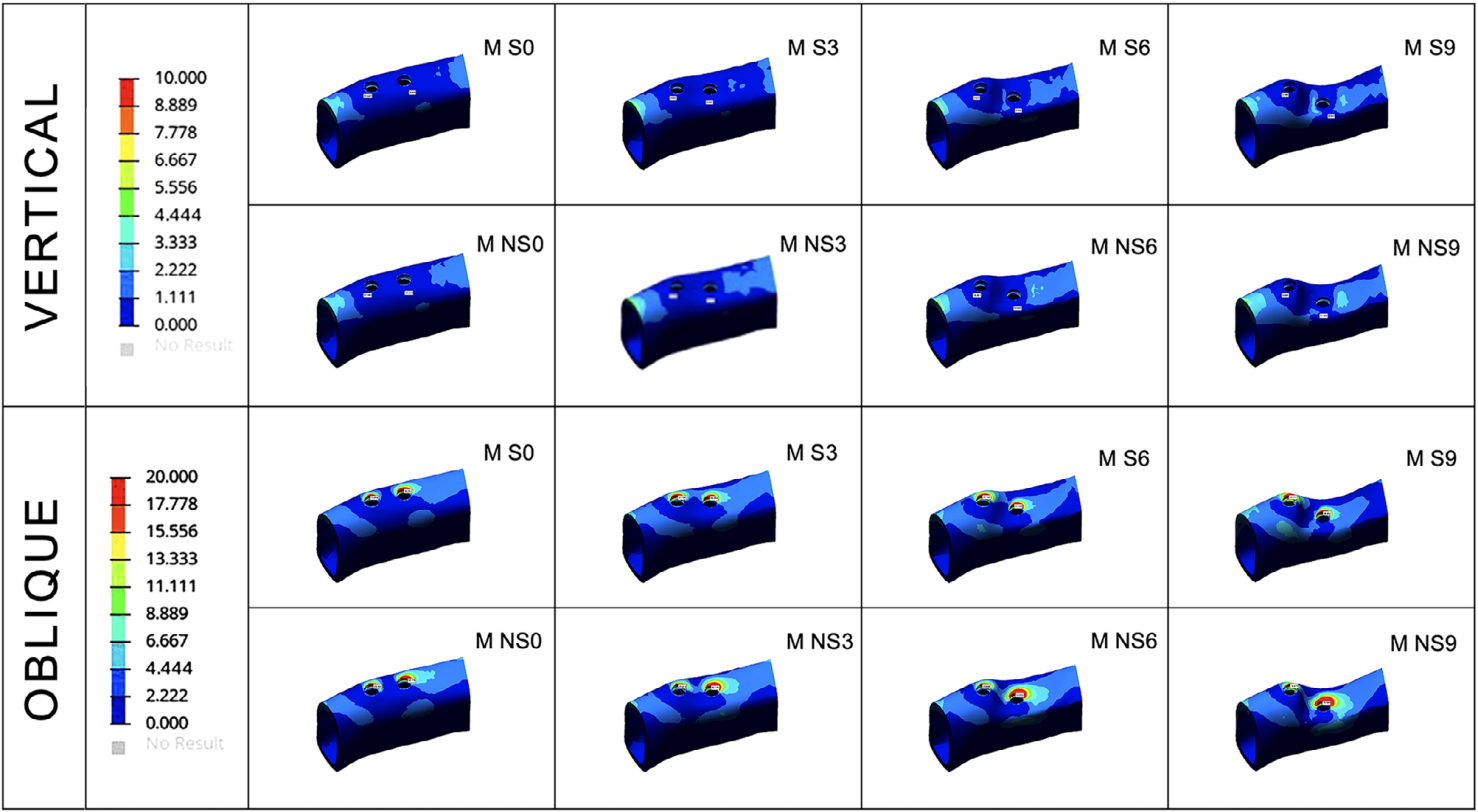
The maximum principal stress in the cortical bone tissue under vertical and oblique loading. (M S0) Splinted and 0 mm bone resorption, (M S3) splinted and 3 mm bone resorption, (M S6) splinted and 6 mm bone resorption, (M S9) splinted and 9 mm bone resorption. (M NS0) Nonsplinted and 0 mm bone resorption, (M NS3) nonsplinted and 3 mm bone resorption, (M NS6) nonsplinted and 6 mm bone resorption, (M NS9) nonsplinted and 9 mm bone resorption.

**FIGURE 6:**
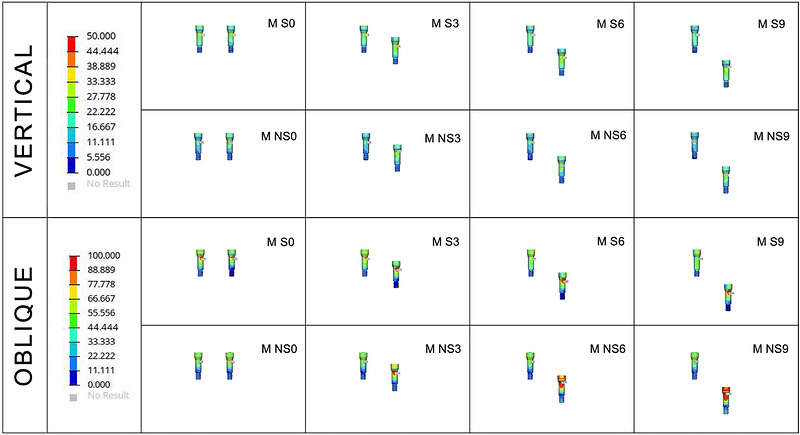
The von Mises stress distribution on abutment screw under vertical and oblique loading. (M S0) Splinted and 0 mm bone resorption, (M S3) splinted and 3 mm bone resorption, (M S6) splinted and 6 mm bone resorption, (M S9) splinted and 9 mm bone resorption. (M NS0) Nonsplinted and 0 mm bone resorption, (M NS3) nonsplinted and 3 mm bone resorption, (M NS6) nonsplinted and 6 mm bone resorption, (M NS9) nonsplinted and 9 mm bone resorption.

Under oblique forces, as the amount of resorption increased, the von Mises stresses were observed to be concentrated at the implant neck of the first molar site for the splinted crowns but distributed to the middle third of the implant from the implant neck for the nonsplinted crowns (Figure 3). For the abutment, in all nonsplinted models, the von Mises stress distribution area gradually expanded in the region below the abutment finish line as the amount of resorption increased (Figure 4). While the maximum principal stress was concentrated on the buccal region of the bone where both implants were placed in splinted models, it was concentrated on the buccal and distal region as the amount of resorption increased in the nonsplinted models (Figure 5). In both splinted and nonsplinted models, von Mises stress values were concentrated at the neck of the abutment screw of the first molar site implant. This concentration was clearly observed in the nonsplinted models (especially in Model NS6 and Model NS9) (Figure 6).

The stress distribution in the first molar site was evaluated in models with similar bone levels under oblique force; when Model S0 was compared with Model NS0, there was an increase in implant, abutment, and bone stress values in Model NS0, while the stresses in the abutment screw decreased. When Model S3 was compared with Model NS3, there was a slight increase in the stress distribution in all components of Model NS3. When Model S6 was compared with Model NS6, there was an increase in stress distribution in all components of Model NS6. When Model S9 was compared with Model NS9, a significant increase in stress distribution in all components was observed in Model NS9 (Tables 3 and  4).

When the von Mises and maximum principal stress distribution at the second premolar site with the same bone level for each model under oblique force was evaluated, the stress values occurring in the implant, abutment and bone decreased in the nonsplinted models compared to the splinted models. Only in the abutment material, von Mises stress values increased in nonsplinted models compared to splinted models. In addition, for models with 9 mm resorption, an increase in von Mises stress was observed in the abutment screw in the nonsplinted model compared to the splinted model. This is an exception for the abutment screw (Tables 3 and  4).

After vertical force application, stress decreased in all components except the abutment screw in both splinted and nonsplinted models at both second premolar and first molar sites with the increase in bone loss (Tables 3 and 4). However, in splinted models at the first molar site, the stress was homogeneously distributed in the platform of the implant; while in nonsplinted models, it was concentrated in the mesial region of the implant with the increase in bone loss (Figure 3). Vertical forces resulted in smaller stress values than oblique forces in all components as in previous studies.^16^, ^28^ In addition, as the amount of resorption increased, oblique forces caused a more significant difference in stress values in bone and implant components than vertical forces.

In the present study, similar to previous studies, the maximum and minimum principal stress values were lower than the maximum and minimum principal stress values in cancellous bone.^31^, ^32^ Therefore, the results were interpreted based on the maximum and minimum principal stress values in cortical bone.

## DISCUSSION

In the present study, the von Mises maximum and minimum principal stresses induced in bone and prosthetic components by splinted and nonsplinted superstructures of two adjacent implants with different bone levels were evaluated using eight different models. The choice of the mandibular second premolar and first molar site in the study was made considering the higher incidence of prosthetic complications such as early tooth loss, bone loss, and screw loosening after implant placement.^33^, ^34^, ^35^

According to the results of the present study, it was observed that the splinted design was more advantageous in terms of stress distribution in bone, implant, and prosthetic components than the nonsplinted design. Also, it was found that increasing the CHS under oblique forces for both prosthetic designs had a stress‐increasing effect. This effect of CHS on stress distribution was more severe in the nonsplinted models. Therefore, the null hypothesis that the CHS and splinting would not affect the stresses measured at bone, implant, and abutment screw was rejected.

It has been previously reported that oblique force application better mimics occlusal loads; however, in some previous studies, forces were applied both vertically and obliquely.^24^, ^36^, ^37^, ^38^, ^39^, ^40^ Therefore, in the present study, a force of 400N (200N × 2) was applied vertically to the central fossae of two adjacent implant crowns and obliquely (30° angle) to the lingual slopes of the buccal cusps. In line with the results of other studies, the stress in cortical bone was higher than in cancellous bone.^31^, ^32^ Stresses were concentrated in the buccal region due to the fact that the forces were applied on the buccal half of the occlusal surfaces of the crown.

There are previous studies evaluating splinted and nonsplinted restorations and restorations with different CIRs.^26^, ^41^, ^42^ The stress around the implant was evaluated in splinted restorations with different CIRs (1/1, 1.5/1, and 2/1) in which two implants were placed in the second premolar and first molar site in the mandible. Similar to the current study, it was stated that increasing CIR increased the stress around the implant. The study also emphasized that increasing CHS was a more effective factor than CIR.^26^ In another study, a 3‐unit restoration on implant was modeled in an edentulous posterior mandible, and it was concluded that splinting the implants had a positive effect on the magnitude and distribution of the stresses similar to the current study.^41^ In some long‐term clinical studies, there was no statistical difference in bone loss and complications around splinted and nonsplinted implants.^12^, ^42^

When designing the current study, the results of a previous study that reported increased biomechanical issues when the crowns were taller than 15 mm were taken into consideration; one of the groups was designed to have CHS taller than 15 mm.^10^ However, in contrast to Nissan's study, there was no significant difference in the von Mises stresses in the prosthetic components between the 14 mm CHS and 17 mm CHS.

In an FEA study, it was concluded that splinted restorations had better stress distribution in the implant and other components despite the varying CIR. The study also reported that implant height had no effect on stress distribution.^28^ Another FEA study on short implants concluded that the effect of implant height and CIR on the stress in the marginal bone was insignificant, while increased CIR was more critical.^43^ Moreover, there are also clinical studies on short implants that emphasized the importance of crown height and showed that an increase in crown height increased the marginal bone loss.^44^ However, other studies evaluating different CIRs have shown that increased CIR is not directly related to increased marginal bone loss and does not constitute a biomechanical risk factor for prosthetic stability and survival of dental implants.^45^, ^46^

A study examining the effect of increased crown height on a single crown concluded that increasing the crown height increased the minimum and maximum principal stress values in the bone, which is in line with the present study findings. However, the results of the study differed from those in the present study in that the von Mises stress in the abutment screw decreased with increased crown height.^23^

Metal‐ceramic, zirconia‐ceramic, monolithic zirconia, and composite‐based materials were used as prosthetic materials in previous studies. It has been shown that the materials used do not differ in terms of the stress on the bone and implant structure.^47^ However, the success of monolithic zirconia restorations in the posterior region against mechanical forces and its advantage in terms of bacterial retention are important reasons for their preference, especially in the last decade; therefore, monolithic zirconia was preferred as the prosthetic material in the present study.^48^

The increased CHS may lead to increased crown‐to‐titanium base ratio, which can affect the bond strength between these components. Previous in vitro studies investigated the effect of titanium base height on the bond strength and performance of the crown‐titanium base complex under loading. The outcomes of these studies suggest a tendency for lower bond strength when the titanium base height is smaller. However, the effect of height should be interpreted considering the titanium base geometry, crown material, and luting cement.^49^, ^50^, ^51^, ^52^ Controlled clinical trials should be performed to better understand the sole effect of titanium base height on the performance of crowns luted on titanium bases.

Considering the findings of the present study and previous study findings, oblique forces have a more destructive effect than vertical forces. Therefore, it can be considered that changes in crown morphology and directing the forces vertically to the implant axis as much as possible can help reduce the stresses and increase the treatment success.

When there is a bone level difference between two adjacent implants, splinting of the restorations reduces the stresses on the bone and implant components. Therefore, splinting of two implants with a clinical bone level difference may be predicted to increase the success of implant treatment. In the absence of a difference in bone level between two adjacent implants, there was no significant difference in bone and implant components between splinted and nonsplinted models. Therefore, the clinical performance of crowns on two adjacent implants with the same bone level whether splinted or nonsplinted, may be similar, however, their performance should be evaluated with controlled in vitro and clinical studies. In this respect, the arrangement and findings of the present study may help in designing and interpreting future studies.

The high abutment screw stresses in nonsplinted crowns suggest that splinting the crowns may reduce screw loosening, one of the most common prosthetic complications in the scenario simulated. However, considering that the FEA study was designed under ideal conditions, and it is only an initial simulation of a clinical situation, the findings of this study should be corroborated with in vitro and clinical studies and long‐term follow‐ups.

## CONCLUSIONS

Crown height was the most important factor for the amount of stress on bone and implant components. Oblique forces produced more stresses on the bone and implant components compared with vertical forces. For two adjacent implants surrounded with similar bone levels, whether the restoration is splinted or nonsplinted may not have a significant effect on stress reduction in the implant complex. However, when the crown height difference between two adjacent implants increases due to bone resorption splinting can be considered as it resulted in decreased von Mises, maximum and minimum principal stresses.

## CONFLICT OF INTEREST STATEMENT

The authors declare no conflicts of interest.
